# 

**Published:** 1945

**Authors:** 


					Vol. LXII. No. 224.
T- B.
^OfVir.
AUTUMN, 1945.
V:
The Bristol
edico-Chirurgical Journal
PUBUSHED UHDBB THE AUSPICES OP
THE BRISTOL MEDICO-CHIRURGICAL SOCIETY.
jt ' ?"?' \ , V ' '..V :
4 , Editor; J. A. NIXON, C.M.G., M.D., F.R.C.P.
& wJtC WITH WHOM ABE ASSOCIATED
a^k 2 3 1946 A' ILES' 0,B*E" M,B" B,s" RR-as-
U n ? I A. RE^bLE SHORT, M.D., B.S., B.Sc., F.R.C.S. ;
{ -\f:* v w. melville capper, m.b., b.s., f.R;C.s.
E. WAISON-WILLIAMS, M.C., Ch.II., F.R.C.S., Assistant Editor.
W;
BRISTOL:
J. W. ARROWSMITH LTD., QUAY STREET & SMALL STREET
5 ?-v^". _? '? ? y>r' -- ? '? '? . g - ' ' * ' . w
Price Three Shillings net.
*# X gJJ'-to. "?* ?
^ ' 11 y - 7? ' ?' ? V
j<r, .* . . ' * ? ' " , 7. ' ? . >' ?'"'<??/ ? r
ISsfiP1'' ?' -v ?Ae ; f ? V? *'?"{ ' 1. ? ,

				

